# Fatal *Mycobacterium colombiense*/cytomegalovirus coinfection associated with acquired immunodeficiency due to autoantibodies against interferon gamma: a case report

**DOI:** 10.1186/1471-2334-13-24

**Published:** 2013-01-22

**Authors:** Sébastien Poulin, Claude Corbeil, Mélanie Nguyen, Anik St-Denis, Lise Côté, Françoise Le Deist, Alex Carignan

**Affiliations:** 1 Department of Microbiology and Infectious Diseases, Centre Hospitalier Universitaire de Sherbrooke, 3001, 12ème Avenue Nord, Sherbrooke, Quebec, J1H 5N4, Canada; 2 Department of Respirology, Hôpital Charles LeMoyne, Greenfield Park, Longueuil, Quebec, Canada; 3 Department of Rheumatology and Allergy, Hôpital Charles LeMoyne, Greenfield Park, Longueuil, Quebec, Canada; 4 Laboratoire de santé publique du Québec (LSPQ), Sainte-Anne-de-Bellevue, Quebec, Canada; 5 Department of Microbiology and Immunology, Department of Pediatrics and Centre de recherche du CHU Sainte-Justine, Université de Montréal, Montreal, Quebec, Canada

**Keywords:** Atypical mycobacteria, Acquired immunodeficiency, Autoantibody, Interferon-gamma, Cytomegalovirus, *Mycobacterium colombiense*

## Abstract

**Background:**

Reports of acquired immunodeficiency due to autoantibodies against interferon gamma in the adult population are increasing. The interleukin-12-dependent interferon-gamma axis is a major regulatory pathway of cell-mediated immunity and is critical for protection against a few intracellular organisms, including non-tuberculous mycobacteria and *Salmonella* spp. We report the first case of a fatal disseminated *Mycobacterium colombiense*/cytomegalovirus coinfection in an adult woman associated with the acquisition of autoantibodies against interferon-gamma.

**Case presentation:**

A 49-year-old woman, born to nonconsanguineous parents in Laos, but who had lived in Canada for the past 30 years, presented with a 1-month history of weight loss, fatigue, cough, and intermittent low-grade fever. A thoracic computed tomography scan revealed an 8 × 7 cm irregular mass impacting the right superior lobar bronchus along with multiple mediastinal and hilar adenopathies. On the fourth day of admission, the patient developed fever with purulent expectorations. Treatment for a post-obstructive bacterial pneumonia was initiated while other investigations were being pursued. Almost every culture performed during the patient’s hospitalization was positive for *M. colombiense*. Given the late presentation of symptoms - at the age of 49 years - and the absence of significant family or personal medical history, we suspected an acquired immunodeficiency due to the presence of anti-interferon-gamma autoantibodies. This was confirmed by their detection at high levels in the plasma and a STAT1 phosphorylation assay on human monocytes. The final diagnosis was immunodeficiency secondary to the production of autoantibodies against interferon-gamma, which resulted in a post-obstructive pneumonia and disseminated infection of *M. colombiense*. The clinical course was complicated by the presence of a multiresistant *Pseudomonas aeruginosa* post-endobronchial ultrasound mediastinitis, cytomegalovirus pneumonitis with dissemination, and finally, susceptible *P. aeruginosa* ventilator-associated pneumonia with septic shock and multiple organ failure, leading to death despite appropriate antibacterial and anti-mycobacterial treatment.

**Conclusions:**

Although rare, acquired immunodeficiency syndromes should be considered in the differential diagnosis of patients with severe, persistent, or recurrent infections. Specifically, severe non-tuberculous mycobacteria or *Salmonella* infections in adults without any other known risk factors may warrant examination of autoantibodies against interferon-gamma because of their increasing recognition in the literature.

## Background

Disseminated non-tuberculous mycobacteria (NTM) infections mainly occur in individuals with impaired cell-mediated immunity, which may result from human immunodeficiency virus (HIV) infection, malignancy (especially hematologic cancers), the use of immunosuppressive drugs, and, more rarely, primary T-cell deficiencies [1]. The interleukin-12 (IL-12)-dependent interferon-gamma (IFN-γ) axis is a major regulatory pathway of cell-mediated immunity and is critical for protection against some intracellular organisms, including NTM and *Salmonella* spp. [2]. Genetic defects in this axis among the pediatric population, producing Mendelian susceptibility to mycobacterial diseases (MSMD), are well recognized [2,3]. However, since 2004, reports of severe, persistent, or recurrent infections with NTM secondary to acquired autoantibodies to IFN-γ have been increasingly encountered among the adult population [4]. In this study, we report the first case of a fatal disseminated *Mycobacterium colombiense*/cytomegalovirus coinfection in an adult woman associated with the acquisition of autoantibodies against IFN-γ.

## Case presentation

**Figure 1:**
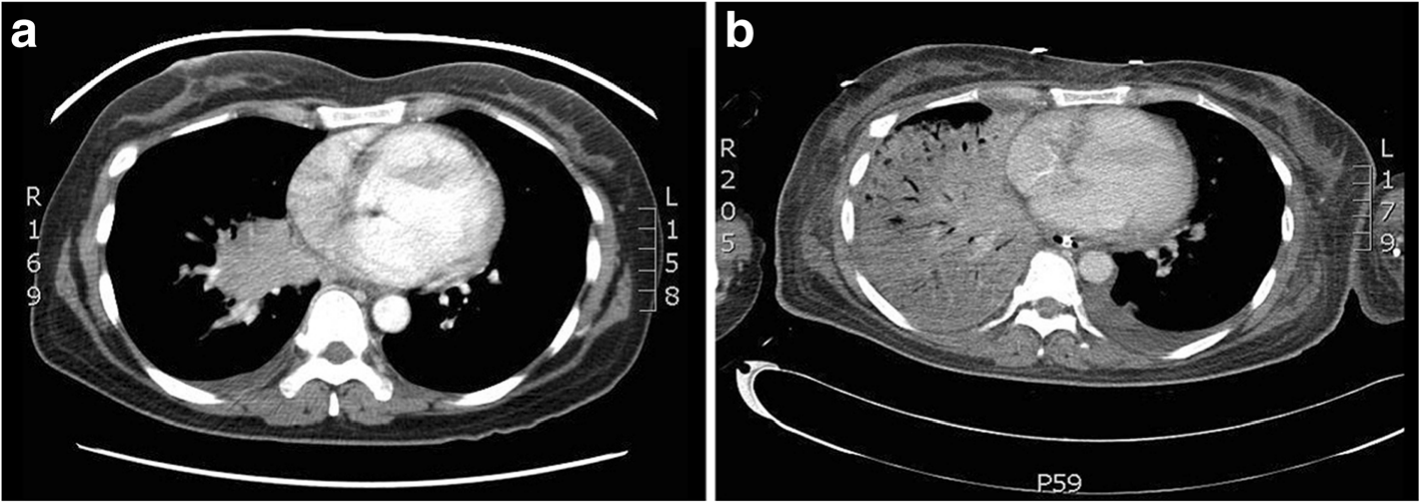
**a Thoracic CT scan at presentation.** Irregular mass impacting the right superior lobar bronchus. **b** Thoracic CT scan on the eighth day in the ICU. Extensive right pulmonary consolidation.

A 49-year-old woman, born to nonconsanguineous parents in Laos, but who had lived in Canada for the past 30 years, presented in May 2010 with a 1-month history of weight loss, fatigue, cough, and intermittent low-grade fever. Her previous family and personal medical history were unremarkable. She could not recall being in contact with tuberculosis. Given that a 7-day course of levofloxacin treatment for presumed right lung pneumonia did not improve her symptoms, she was subjected to further examinations. An initial bronchoscopy showed friable, bleeding, and irregular mucosa of the right upper lobe entry. Biopsies were not feasible, but Ziehl-Neelsen and auramine stains of the bronchoalveolar lavage (BAL) were negative and cytology was normal. A thoracic computed tomography (CT) scan revealed atelectasis caused by an 8 × 7 cm irregular mass impacting the right superior lobar bronchus along with multiple mediastinal and hilar adenopathies (Figure 1a).

Since the most probable diagnosis was a neoplastic process, the patient was discharged home to await a scheduled transthoracic biopsy (TTB). However, when a previous BAL culture was found to be positive for acid-fast bacilli (AFB), she was readmitted to rule out active tuberculosis. Upon presentation, she was afebrile and her vital signs were normal. There was no regional adenopathy, and the physical examination was normal except for reduced vesicular murmur sounds on auscultation over the right lung. The peripheral white blood cell count was 26 × 10^9^/L, with 21 × 10^9^/L neutrophils and 3 × 10^9^/L lymphocytes. The inflammatory syndrome was considered important due to a serum C-reactive protein concentration of 111 mg/L and erythrocyte sedimentation rate of 25 mm/h. The procalcitonin concentration was 19 μg/L. The hemoglobin level was 71 g/L with a mean corpuscular volume of 73 fL, and the platelet count was at 766 × 10^9^/L. Other routine biochemical tests were normal. Polymerase chain reaction (PCR) performed 3 days after admission, using morning sputum, was negative for *Mycobacterium tuberculosis* complex. On the fourth day, the patient developed fever with purulent expectorations. While other investigations were being pursued, treatment for post-obstructive pneumonia was initiated with piperacillin-tazobactam.

An endobronchial ultrasound (EBUS) revealed purulent secretions and a diminished right superior lobar bronchus caliber. Multiple transbronchial needle aspirations of adenopathies were also performed. Two days later, the patient’s condition necessitated her transfer to the intensive care unit (ICU), and vancomycin was added. The peripheral white blood cell count was 47 × 10^9^/L and a chest X-ray film showed a new infiltration process affecting the right lower lobe and median lobe with an important pleural effusion. Analysis of the pleural fluid showed an uncomplicated exudative effusion (pH >7.2) with a predominance of lymphocytes. On the fourth day in the ICU, endotracheal intubation and support with amines were required for septic shock. The results of mediastinoscopy biopsies followed by a TTB of the mass lesion were non-diagnostic, showing only extensive fibrosis and unspecific acute and chronic reactive lymphoid infiltrates. Granuloma formation was not observed.

On the eight day in the ICU, the first preliminary report of the initial BAL sample revealed that it was positive for *M. colombiense*, as shown by analyzing genomic deletions and rRNA 16S gene sequencing (Quebec Public Health Laboratory). As radiologic control had also deteriorated (Figure 1b), the decision to commence treatment on the same day with rifampicin, clarithromycin, and ethambutol was taken. Later sensitivity analysis by broth microdilution revealed that *M.colombiense* was susceptible to clarithromycin with a minimum inhibitory concentration (MIC) of 0.25 mg/L. Rifampin's MIC was 4 mg/L and ethambutol's MIC was >16 mg/L. Although ethambutol and rifampin are useful clinically in cases of *Mycobacterium avium complex* infections [5], breakpoints for determining susceptibility and resistance have not been established by the Clinical and laboratory standards institute M24-A2 (CLSI M24-A2) [6]. HIV tests were repeatedly negative. Two days later, multiple organ failure (MOF) developed. Since the previous mediastinoscopy culture was positive for a multiresistant *Pseudomonas aeruginosa* strain (Table 1), vancomycin was stopped and piperacillin-tazobactam was changed to doripenem and tobramycin. On the patient’s 27th day in the ICU, administration of anti-*Pseudomonas* antibiotics was terminated because her clinical condition was stable. However, when 2 tracheal aspirates were found to be positive for susceptible *P. aeruginosa* (Table 1) and clinical deterioration was again noted, treatment with piperacillin-tazobactam and ciprofloxacin was started. Unfortunately, the patient died from septic shock with MOF on the 34th day in the ICU.

**Table 1:** Culture specimens during the patient’s hospitalization

**Specimen**	**Date**	**Auramine**	**NTM**	**Other**
BAL	7/6/10	-	*M. colombiense*	-
Sputum	27/6/10	-	*M. colombiense*	-
Sputum	28/6/10	1–9/100	*M. colombiense*	-
EBUS	30/6/10	-	*M. colombiense*	-
Sputum	2/7/10	-	-	*C. albicans*
Mediastinoscopy	6/7/10	-	*M. colombiense*	*P. aeruginosa*
Tracheal aspirate	8/7/10	1–9/100	*M. colombiense*	-
Tracheal aspirate	9/7/10	1–9/100	*M. colombiense*	-
Tracheal aspirate	10/7/10	1–9/100	*M. colombiense*	-
Right pleural effusion	11/7/10	-	*M. colombiense*	-
Bronchial lavage	18/7/10	1–2/100	*M. colombiense*	-
Bronchial lavage	26/7/10	-	*M. colombiense*	-
Bronchial lavage	29/7/10	-	-	CMV
Tracheal aspirate	31/7/10	-	-	*P. aeruginosa*
Tracheal aspirate	2/8/10	-	*M. colombiense*	*P. aeruginosa*

**Figure 2:**
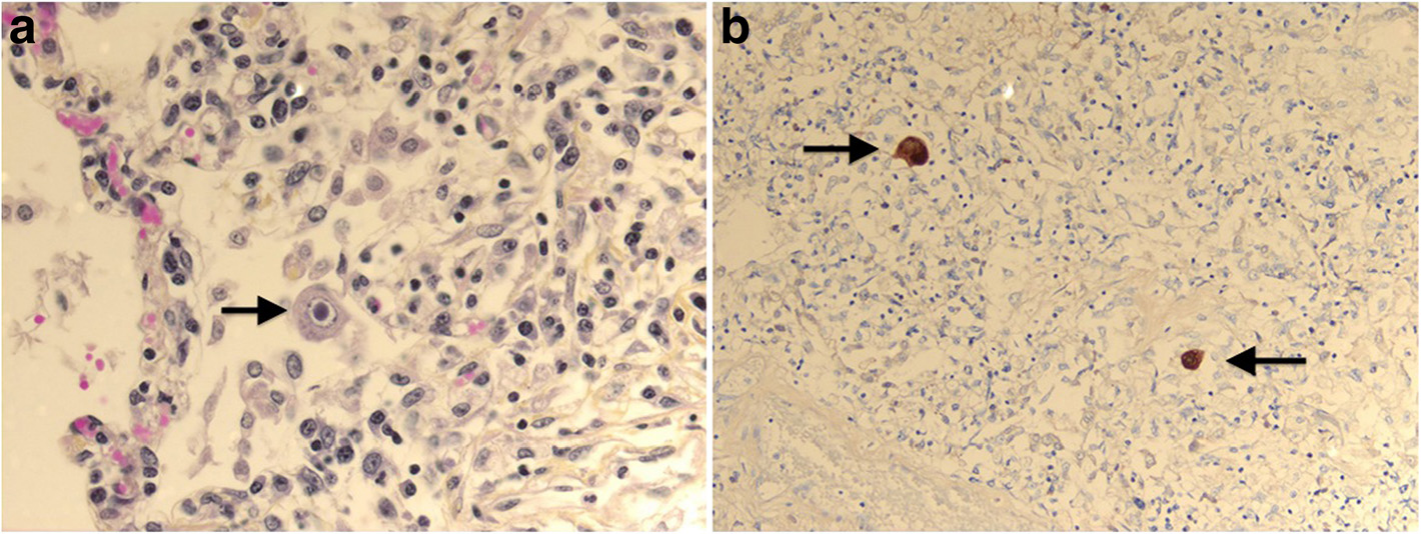
**a CMV inclusion.** Histological analysis of the lung reveals a very large cell that displays a large violet intranuclear inclusion with a small clear halo (typical owl-eye inclusion; arrow). **b** CMV immunohistochemistry. Immunohistochemical analysis of the lung reveals anti-CMV positive cells (arrows).

Interestingly, almost every culture performed during the patient’s hospitalization was positive for *M. colombiense* (Table 1). Although cytomegalovirus (CMV) serology was not performed for this patient before her death, samples from the final bronchoscopy grew CMV in fibroblast cell culture many days later (Table 1). The pathologist also identified CMV inclusions in the lungs, mediastinal adenopathies, thyroid gland, pancreas, and adrenal glands (Figure 2a). This observation was also confirmed by immunohistochemistry (Figure 2b).

Post-mortem examination of the right lung revealed an organizing pneumonia, and the mediastinum contained a lot of adherence, fibrosis, and adenopathies with lymphoid hyperplasia. No granuloma formation was detected in the lungs or in any of the other organs examined. An immunologic investigation performed before the death of our patient revealed normal counts of lymphocyte populations (CD4+, CD8+, CD19+, CD56+) and normal levels of immunoglobulins. We concluded that a genetic immunological deficit was very unlikely because of the late presentation of symptoms—at the age of 49 years—and the absence of significant family or personal medical history. Instead, we suspected an acquired deficit; thus, we tested for the presence of anti-IFN-γ autoantibodies in the patient’s plasma. The IFN-γ titer—defined as the dilution of a patient’s plasma that inhibits 50% fixation of 125 pg/mL IFN-γ in a standard IFN-γ ELISA assay (eBioscience ELISA kit)—was positive at 1:53725. Therefore, the final diagnosis was immunodeficiency secondary to the production of autoantibodies against IFN-γ, which resulted in post-obstructive pneumonia caused by lymphadenitis and disseminated infection of *M. colombiense*. The clinical course was complicated by the presence of multiresistant *P. aeruginosa* infection post-EBUS mediastinitis, CMV pneumonitis with dissemination, and finally, susceptible *P. aeruginosa* ventilator-associated pneumonia with septic shock and MOF leading to death.

## Discussion

A total of 29 cases of disseminated NTM infections secondary to the production of autoantibodies against IFN-γ had been reported [1,2,7,8] before the recent large prospective study by Browne et al. [9]. Compared to control groups, neutralizing anti-IFN-γ autoantibodies was associated with disseminated NTM or other different opportunistic infections among more than 80 patients from Thailand or Taiwan. In these studies, the organs involved included the lymph nodes, bone marrow, bones, lungs, skin, and soft tissues. In contrast to the case reported here, most of them described the formation of complete or poorly formed granulomas. However, several different forms of MSMD are associated with the absence of granuloma formation [10,11], suggesting that our observations may be plausible. Positive blood cultures were infrequently noted [1]. The presence of both rapidly growing NTMs (e.g., *M. chelonae*, *M. fortuitum*, and *M. abscessus*) and slowly growing NTMs (e.g., *M. avium* complex (MAC), *M. szulgai*, *M. kansasii*, *M. scrofulaceum*, and *M. intermedium*) has been described, and the most frequently detected are MAC and *M. chelonae*. Notably, to our knowledge, our case is the first to specifically identify *M. colombiense*. In fact, *M. colombiense* is a novel member of the *Mycobacterium avium complex* (MAC) that was first isolated in 2006 from blood specimens of four HIV-positive patients in Colombia, South America [12]. Interestingly, only 3 reports have shown its capability of causing lymphadenitis, and they were from cervical [13,14] or subclavicular lymph nodes [15]. It was also isolated only twice from respiratory specimen [14]. Previous identifications of MAC organisms often relied on hybridization techniques, which hampered distinguishing between the various MAC species compared to current sequence-based identifications and genotyping [14]. Because no consensus about the preferred anti-mycobacterial regimen exists yet for *M. colombiense*, we followed current disseminated *Mycobacterium avium complex* infections guidelines using clarithromycin with rifampin and ethambutol [5].

Pathogens other than NTM or *Salmonella* spp. have been reported as coinfecting organisms in patients with acquired autoantibodies against IFN-γ. Namely, these include *M. tuberculosis*, varicella-zoster virus, *Burkholderia cocovenenans*, *Candida*, *Aspergillus*, *Achromobacter* spp*.*, *Enterococcus* spp., *Streptococcus pyogenes, Penicillium marneffei, Strongyloides stercoralis, Cryptococcus neoformans and Histoplasma capsulatum*[1,2,8,9]. However, the direct involvement of these coinfecting organisms in the defect in the IL-12―dependent IFN-γ axis is not entirely clear. Moreover, *Pseudomonas* spp. have only been described once in coinfection [16]. Since our patient had several other risk factors for *P. aeruginosa* infection (e.g., ICU, endotracheal intubation, and antibiotics) and because *Pseudomonas* is not an intracellular pathogen, it seems unlikely to be a direct consequence of a defect in the IL-12―dependent IFN-γ axis. Post-mortem serum sample was collected to measure CMV-specific immunoglobulin G (CMV-IgG) and CMV-specific immunoglobulin M (CMV-IgM) markers. As our patient was IgM negative, and IgG positive, we assumed that the CMV infection was most likely a reactivation process. Prior to our report, CMV coinfection had only been described in 1 case [4] of acquired autoantibodies against IFN-γ. However, cases of CMV infection have been described in MSMD [3,17]. Therefore, it is possible that the IL-12―dependent IFN-γ axis, strongly diminished by autoantibodies in our patient, played a significant role in her disseminated CMV infection.

**Figure 3:**
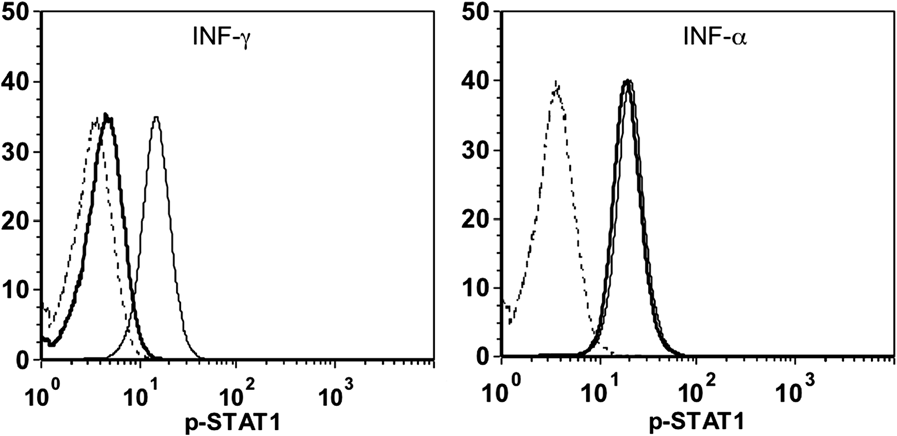
**Inhibition of INF-γ-induced STAT1 phosphorylation in control monocytes by patient’s plasma.** Fluorescence histogram displays STAT1 phosphorylation gated on CD14+ (monocytes) population. Whole blood samples were incubated without (−−--) or with INF-γ (1000 IU/ml)(left panel), or with INF-α (1000 IU/ml) (Right panel) pre-mixed with patient plasma (—) or control plasma (—).

Similar to the patient described in the present study, cases of acquired autoantibodies against IFN-γ have mainly been reported in Asian female patients [1]. Nonetheless, this is the first reported case from Laos. The high incidence rate in Asian populations may be associated with an unknown HLA allele variant [2]. In contrast to what is sometimes observed, our patient did not present any known autoimmune endocrinopathy or reactive skin diseases such as Sweet’s syndrome or pustular psoriasis [1,2]. Given the severe presentation of our case and the high titer of autoantibodies detected, we hypothesize that such autoantibodies would produce a strong inhibitory effect against IFN-γ signal transduction and IFN-γ―dependent upregulation of TNF-α and IL-12, as was previously shown by Patel *et al.*[16]. Naturally occurring anti-IFN-γ autoantibodies have been previously described in cases of viruses and mycobacteria infections. However, they usually occurred at low titers and were biologically inactive [16,18]. To confirm our hypothesis, we performed a STAT1 (implicated in the IFN-γ―dependent upregulation of TNF-α and IL-12 [19]) phosphorylation assay using FACSCanto flow cytometry (BD Biosciences) on preserved plasma sample (Figure 3). As expected, phosphorylation of STAT1 in human monocytes after stimulation with INF-γ was inhibited by the patient's plasma, but not when the monocytes were stimulated with IFN-α. We also tested HIV serology twice, and found the immunologic screening to be normal. Since the patient was not known to have any other risk factor for depressed cell-mediated immunity, it is likely that our diagnosis is accurate.

Outcomes in cases of acquired autoantibodies to IFN-γ with NTM infections in the adult population range from fatal infection to complete recovery. These cases frequently result in refractory infection with recurrent or relapsing episodes, despite aggressive and prolonged treatment with 3 or more anti-mycobacterial agents. Additional adjunctive therapies—such as plasmapheresis, intravenous immunoglobulins, exogenous IFN-γ, and cyclophosphamide followed by corticotherapy—have been reported with varying rates of success. In a recent study by Browne *et al.*[7], 4 cases of refractory disease treated with rituximab showed clinical, radiological, and laboratory evidence of therapeutic improvement, including improved IFN-γ signaling and reduction in anti-IFN-γ autoantibody levels. The rare fatal cases [4,8,20] described by Browne and colleagues were infections that did not respond to months of anti-mycobacterial therapy. Therefore, we believe that our patient is the first to present a fulminant evolution, which led to death in her first hospitalization. This may be explained by the stepwise addition of severe *Pseudomonas* and disseminated CMV coinfections in a patient already weakened by NTM infection. Despite early initiation (on the same day as the preliminary mycobacterial culture result was known) of anti-mycobacterial treatment based on current recommendations for *Mycobacterium avium complex* disseminated infections, it is likely that the other coinfections had progressed significantly enough to cause mortality.

### Staining of blood samples for STAT1 phosphorylation

One hundred microliter (μl) of normal heparinized whole blood were incubated or not either with 20μl of IFN-γ (10000 IU/ml) or IFN-α (10000 IU/ml) premixed with patient plasma or control plasma (V/V:1/1) for 15 minutes at 37°C. Erythrocytes were lysed with BD phosflow™ Lyse/Fix Buffer (1X) for 10 minutes at 37°C., After surface staining with FITC-conjugated anti-CD14 antibody, cells were permeabilized with BD phosflow™ Perm Buffer III, followed by intracellular staining with Alexa 647-conjugated antibody recognizing pSTAT1 (Y701). Data acquisition was performed by FACSCanto flow cytometry (BD Biosciences) and analysed with FCS Express 4 Flow Research Edition. Fluorescence histogram displays STAT1 phosphorylation gated on CD14+ population (Figure 3).

## Conclusions

This case demonstrates that, although rare, primary or acquired immunodeficiency syndromes should be considered in the differential diagnosis of patients with severe, persistent, or recurrent infections. Specifically, severe NTM or *Salmonella* infections in adults without any other known risk factors may warrant examination of autoantibodies against IFN-γ. Early recognition is vital in such cases in order to consider suitable treatment options, including, for example, the use of rituximab [7,19].

### Consent

Written informed consent was obtained from the next of kin of the patient for publication of this case report and any accompanying images. A copy of the written consent is available for review by the Series Editor of this journal.

## Abbreviations

NTM: Non-tuberculous mycobacteria; INF-γ: Interferon-gamma; MSMD: Mendelian susceptibility to mycobacterial diseases; BAL: Bronchoalveolar lavage; TTB: Transthoracic biopsy; AFB: Acid-fast bacilli; EBUS: Endobronchial ultrasound; MOF: Multiple organ failure; MIC: Minimum inhibitory concentration.

## Competing interests

The authors declare that they have no competing interests.

## Authors’ contributions

SP drafted and wrote the manuscript. CC and MN took care of the patient and contributed to coordinating the manuscript. ASD carried out the immunoassays. LC carried out the microbiological assays. FLD carried out the immunoassays and contributed to coordinating the manuscript. AC contributed to the draft of the manuscript and revised the manuscript. All authors have read the manuscript and approved its final version.

## Pre-publication history

The pre-publication history for this paper can be accessed here:

http://www.biomedcentral.com/1471-2334/13/24/prepub
